# Sirtuin-5 Is Recruited to Hepatic Peroxisomes in Mice Fed Dodecanedioic Acid but Has Little Impact on the Peroxisomal Succinylome

**DOI:** 10.3390/biom14121508

**Published:** 2024-11-26

**Authors:** Yuxun Zhang, Bob B. Zhang, Sivakama S. Bharathi, Joanna Bons, Jacob P. Rose, Samah Shah, Steven F. Dobrowolski, Sunder Sims-Lucas, Birgit Schilling, Eric S. Goetzman

**Affiliations:** 1 Department of Pediatrics, School of Medicine, University of Pittsburgh, Pittsburgh, PA 15224, USA; 2 The Buck Institute for Research on Aging, Novato, CA 94945, USA; 3 Department of Pathology, University of Pittsburgh School of Medicine, Pittsburgh, PA 15261, USA

**Keywords:** peroxisome, sirtuin-5, fatty acid oxidation

## Abstract

Lysine succinylation, and its reversal by sirtuin-5 (SIRT5), is known to modulate mitochondrial fatty acid β-oxidation (FAO). We recently showed that feeding mice dodecanedioic acid, a 12-carbon dicarboxylic acid (DC_12_) that can be chain-shortened four rounds to succinyl-CoA, drives high-level protein hypersuccinylation in the peroxisome, particularly on peroxisomal FAO enzymes. However, the ability of SIRT5 to reverse DC_12_-induced peroxisomal succinylation, or to regulate peroxisomal FAO in this context, remained unexplored. Here, we showed that feeding DC_12_ strongly recruits SIRT5 into hepatic peroxisomes. Knocking out SIRT5 impaired peroxisomal FAO as evidenced by reduced ^14^C-DC_12_ flux in liver homogenates and elevated levels of partially shortened DC_12_ catabolites in urine. Further, mass spectrometry revealed a trend toward less peroxisomal protein succinylation in SIRT5 knockout liver. This is consistent with a reduced flux of DC_12_ through the peroxisomal FAO pathway, thereby reducing the production of the succinyl-CoA that chemically reacts with lysine residues to produce protein succinylation. Mass spectrometry comparisons of site-level succinylation in wildtype and SIRT5 knockout liver did not reveal any clear pattern of SIRT5 target sites in the peroxisome after DC_12_ feeding. However, SIRT5 co-immunoprecipitated with 15 peroxisomal proteins, including the key peroxisomal FAO enzymes acyl-CoA oxidase-1 and enoyl-CoA/3-hydroxyacyl-CoA dehydrogenase (EHHADH). In vitro, recombinant SIRT5 partially desuccinylated chemically modified recombinants ACOX1a, ACOX1b, and EHHADH. Desuccinylation by SIRT5 had no effect on enzyme activity for ACOX1a and EHHADH. For ACOX1b, SIRT5-mediated desuccinylation decreased activity by ~15%. Possible interpretations of these data are discussed.

## 1. Introduction

Lysine succinylation is a post-translational modification arising from a non-enzymatic reaction between succinyl-CoA and solvent-accessible lysine residues on the surface of proteins [1]. Succinylation has been well-characterized in the mitochondria where it has detrimental effects on protein function if left unchecked [2,3]. Reversing this PTM is handled by sirtuin-5 (SIRT5), an NAD+-dependent lysine deacylase [2]. SIRT5 has an isoform that localizes to the mitochondria, where it desuccinylates, deglutarylates, and demalonylates numerous substrates [3], and an isoform that localizes to the cytoplasm where it demalonylates additional protein substrates [4]. Interestingly, SIRT5 was recently shown to localize to the peroxisome in the hepatic cell lines HepG2 and Huh7 [5]. In the peroxisome, SIRT5 desuccinylated acyl-CoA oxidase-1 (ACOX1), the rate-limiting enzyme of peroxisomal fatty acid β-oxidation (FAO). SIRT5 desuccinylation of ACOX1 destabilized the enzyme to reduce its activity. This suggests that SIRT5 may suppress peroxisomal FAO, being the opposite of its mitochondrial role in promoting FAO.

The observation that SIRT5 desuccinylates ACOX1 raises the question of how lysine succinylation arises within the peroxisomal compartment. Chen et al. [5] postulated that impaired function of mitochondrial succinate dehydrogenase could cause an accumulation of succinate, with subsequent transfer to peroxisomes. In this model, succinate entering peroxisomes would require esterification to coenzyme-A prior to succinylating proteins, as free succinate does not react with lysine residues. The mechanism of peroxisomal succinate esterification was not explored. In contrast, our recent study on dodecanedioic acid suggested a more direct route of succinyl-CoA formation inside the peroxisome [6]. Dodecanedioic acid is a 12-carbon dicarboxylic acid (hereafter, DC_12_), which is a naturally occurring fatty acid in liver and kidney, formed through a poorly understood ω-oxidation pathway. Synthesis of DC_12_ by the ω-oxidation pathway begins in the endoplasmic reticulum with the hydroxylation of lauric acid (C_12_) on the terminal (ω) carbon. Two more steps, catalyzed by an alcohol dehydrogenase and an aldehyde dehydrogenase, complete the conversion of the ω carbon into a carboxyl group, thereby rendering the fatty acid “dicarboxylic”. DC_12_ is preferentially degraded in the peroxisome rather than the mitochondria [7]. It is activated to DC_12_-CoA inside the peroxisome and then chain-shortened through four rounds of peroxisomal FAO to succinyl-CoA [8]. We fed mice a diet enriched in DC_12_ and observed massive increases in peroxisomal protein succinylation, including sites on ACOX1 [6]. We did not, however, interrogate the function of peroxisomal SIRT5 in the context of DC_12_ feeding. The work of Chen et al. suggests that succinylation of ACOX1 during DC_12_ feeding may enhance peroxisomal FAO via increased ACOX1 activity, while the presence of peroxisomal SIRT5 could hinder it. Here, we set out to investigate the role of SIRT5 during DC_12_-induced peroxisomal protein hypersuccinylation.

## 2. Materials and Methods

### 2.1. Animals and DC_12_ Administration

All animal protocols were approved by the University of Pittsburgh Institutional Animal Care and Use Committee (IACUC), and all experiments were conducted in accordance with the guidelines and regulations set forth in the Animal Welfare Act (AWA) and PHS Policy on Humane Care and Use of Laboratory Animals. SIRT5-/- and wildtype control mice were purchased from Jackson Laboratories (Bar Harbor, ME, USA) and bred in-house. The mice were maintained on a 12 h light/dark cycle in a pathogen-free barrier facility. In initial experiments, the DC_12_ diet was made by mixing DC_12_-free acid (Sigma-Aldrich Co., St. Louis, MO, USA) by weight (10% *w*/*w*) into a powdered standard rodent diet. In experiments that monitored the physiological response to DC_12_, including EchoMRI and urine collection, the diet was manufactured as pellets by Research Diets, Inc. (New Brunswick, NJ, USA). This diet contained 51% of calories as carbohydrate, 21% as DC_12_, 12% as soybean oil, and 16% as protein. The isocaloric control diet contained 51% of calories as carbohydrate, 33% as soybean oil, and 16% as protein. For physiological experiments, mice were maintained on these diets for five weeks. Afterward, body composition was determined using the EchoMRI-100 system (Echo Medical Systems, Houston, TX, USA), and overnight urine samples were collected using metabolic caging.

### 2.2. Density Gradient Centrifugation

Organelles were separated by density gradient centrifugation using Optiprep [9,10], with minor modifications that we previously described [11]. Fresh liver tissue (0.3–0.5 g) was washed with cold PBS to remove blood and then minced in Petri dishes on ice. The minced tissue was transferred with 5 mL of cold PBS into ice-cold glass tubes (15 × 85 mm) and homogenized with 10 slow up and down strokes in 5 mL of ice-cold Extraction Buffer (5 mM MOPS, pH 7.65, with 250 mM sucrose, 1 mM EDTA, 0.5% ethanol and 0.2 mM PMSF). The homogenate was centrifuged at 1000× *g* for 10 min to remove nuclei and other cell debris. The supernatant was transferred to a 15 mL centrifuge tube and centrifuged at 2000× *g* for 10 min; the supernatant from this spin was then centrifuged at 25,000× *g* for 20 min. The pellet was resuspended in extraction buffer (0.4 mL per gram of original tissue), diluted with OptiPrep Density Gradient Medium (60% iodixanol, Sigma, D1556) and OptiPrep Dilution Buffer (5 mM MOPS, pH 8.0, with 1 mM EDTA and 0.1% ethanol) to make a diluted sample containing 22.5% OptiPrep. In ultracentrifuge tubes, ¼ volume of 27.5% OptiPrep solution was overlaid with ½ volume of the diluted sample, followed by a ¼ volume of 20% OptiPrep Solution. These layered samples were centrifuged for 3 h at 50,000× *g* or 15 h at 10,000× *g*. A cloudy, floating layer on the top of the gradient was primarily endoplasmic reticulum (ER) and lysosomes, a ring at the 22.5%/27.5% interface was mitochondria, and a slightly floating pellet at the bottom of the tube was pure peroxisome fraction.

### 2.3. SIRT5 Co-Immunoprecipitation

Liver lysate in RIPA buffer with protease inhibitors was centrifuged at 16,100× *g* for 15 min. The supernatant was transferred to a fresh tube and 20 µL Protein A agarose (Santa Cruz Biotechnologies, sc-2001, Santa Cruz, CA, USA) slurry was added to pre-clear lysate for 1 h on a rotating mixer, followed by centrifugation at 1000× *g* for 1 min to pellet the agarose beads. The supernatant was transferred to a fresh tube, and 5 µg of recombinant 6His tagged SIRT5 was added and incubated at 37 °C for 30 min. A total of 50 µL of polyHis-antibody conjugated agarose slurry (Santa Cruz Biotechnologies, sc-803AC) was added and incubated on the rotating mixer overnight at 4 °C. The agarose beads were pelleted by centrifugation at 1000× *g* for 1 min. The beads were washed 4 times with the RIPA buffer (1 mL) and pelleted each time at 1000× *g* for 1 min. A total of 40 µL of 2× Laemmli buffer was added to the resulting beads and heated at 80 °C for 10 min. After centrifugation, the supernatant was used for SDS-PAGE. Bands were excised and submitted to the University of Pittsburgh Health Sciences Mass Spectrometry Core Facility for identification of immunoprecipitated proteins.

### 2.4. Immunoblotting

Tissue lysates were electrophoresed on Criterion SDS polyacrylamide gels (Bio-Rad, Hercules, CA, USA) and transferred to nitrocellulose membranes. Antibodies used were anti-succinyllysine (PTM-401 and PTM-419, PTM Biolabs, Hangzhou, CHN); anti-acyl-CoA oxidase-1 (ACOX1; Abcam #ab59964, Waltham, MA, USA); anti-sirtuin-5 (SIRT5; Cell Signaling #8782, Danvers, MA, USA); anti-heat shock protein-60 (HSP60; Cell Signaling #12165), anti-catalase (CAT; Sigma #C0979, St. Louis, MO, USA); anti-peroxisomal membrane protein-70 (PMP70; Abcam ab85550); and anti- mitochondrial import inner membrane translocase subunit TIM23 (TIM23; BD Biosciences #611222, San Jose, CA, USA). After incubation with HRP-conjugated secondary antibodies, blots were visualized with chemiluminescence (Bio-Rad Clarity family ECL reagents), and in some cases, scanned and subjected to densitometric analysis using ImageJ software version 1.54 h (NIH).

### 2.5. Fatty Acid Oxidation Flux Assays

1-^14^C-labeled lignoceric acid (C_24_) and 1-^14^C-dodecanedioc acid (DC_12_) were sourced from PerkinElmer (Waltham, MA, USA) and Moravek, Inc. (Brea, CA, USA), respectively. Just prior to assay, the fatty acids were dried to completion under nitrogen and then solubilized in 10 mg/mL α-cyclodextrin via incubation at 37 °C for 30 min [12]. Each was used at 0.5 μCi per mL, with a final substrate concentration of 50 µM. Liver homogenate was preprepared by homogenizing ~60 mg of freshly harvested liver in 10 volumes of STE buffer (0.25 M sucrose, 10 mM Tris-HCl, 1 mM EDTA pH 7.4) with 10 up and down passes of a Teflon pestle. The FAO protocol has been described in detail elsewhere [13,14]. Briefly, FAO reactions consisted of ~100 µg of liver lysate in a reaction volume of 200 µL containing 100 mM sucrose, 10 mM Tris-HCl, 5 mM KH_2_PO_4_, 0.2 mM EDTA, 80 mM KCl, 1 mM MgCl_2_, 0.1 mM malate, 0.05 mM coenzyme A, 2 mM ATP, and 1 mM DTT. After 2 h incubation at 37 °C, the reactions were stopped by adding perchloric acid to a 0.5 M final concentration. Following centrifugation to remove precipitated material, the water-soluble ^14^C-labeled FAO products were isolated by methanol-chloroform extraction and quantified on a scintillation counter [15,16]. The results were normalized to protein concentration.

### 2.6. Urinary Dicarboxylic Acids

Urine was collected during the dark cycle (12 h) and subjected to gas chromatography and mass spectrometry (GC-MS) at the Children’s Hospital of Pittsburgh Clinical Biochemical Genetics Laboratory. Creatinine concentration was determined using a kit (Cayman Chemical, Ann Arbor, MI, USA). For each sample, a volume of urine was used that was equal to 1.0 mM of creatinine. A 2-phenylbutyrate internal standard was added and organic acids were extracted by sequential ethyl ether and acetoacetate extractions. After trimethylsilane derivatization, the analysis was conducted on an Agilent 7890A gas chromatograph and 5975C mass spectrometer. All peak areas were normalized to that of 2-phenylbutyrate. Fragmentation patterns of urine analytes were compared to an internally compiled fragmentation library and the fragmentation library of the National Institute for Standards and Technology.

### 2.7. Proteomics and Succinylomics

Pieces of frozen liver (~100 mg) were homogenized, alkylated, trypsinized, clarified, and desalted exactly as described [6]. A total of 100 μg of each peptide elution was set aside for analysis of protein-level changes, and the remaining material was used for the enrichment of succinylated peptides with the PTMScan Succinyl-Lysine Motif Kit (Cell Signaling Technologies). LC-MS/MS analyses were performed on a Dionex UltiMate 3000 system online coupled to an Orbitrap Eclipse Tribrid mass spectrometer (Thermo Fisher Scientific, San Jose, CA, USA). All samples were acquired in data-independent acquisition (DIA) mode. DIA data were processed in Spectronaut (version 14.10.201222.47784) using directDIA for both the protein level as well as PTM enriched samples. Differential expression analysis was performed using a paired *t*-test, and *p*-values were corrected for multiple testing. For whole lysate (protein level) analysis, statistically significant differences were defined as a q-value < 0.01 and an absolute Log_2_ (fold-change) > 0.58. For the PTM analysis, significance was established as q-value < 0.05 and absolute Log_2_ (fold-change) > 0.58. Peroxisomal proteins were mapped using GO Cellular Component identifiers and verified manually by cross-referencing to Peroxisome-Db [17].

### 2.8. Recombinant Protein Expression and Purification

Coding sequences for human SIRT5 and the inactive mutant H158Y were incorporated into the pET28a-LIC plasmid with a 6His tag coding sequence at 5′-end. The plasmids were transformed onto E. coli strain BL21(DE3) competent cells. The cells were cultured at 37 °C in LB medium, and protein expression was induced by adding 0.5 mM IPTG when OD was 0.4. The expression was continued overnight with continuous shaking at 30 °C. Cells were lysed on ice by sonication in lysis buffer (50 mM NaPO_4_, 300 mM NaCl, 10 mM imidazole, pH 8.0). Ultracentrifugation was employed to separate lysate supernatant and cell debris. The recombinant proteins were purified on HisTrapTM HP columns (Cytiva Sweden AB) with an AKTA Pure system. The washing buffer was 50 mM NaPO_4_, 300 mM NaCl, 10% glycerol, 20 mM imidazole, pH 8.0. The proteins were eluted with an imidazole gradient.

Human ACOX1a and ACOX1b coding sequences were incorporated into pET21a(+) plasmids with a 6× His tag coding sequence at 5′-end following a start codon. The plasmids were transformed onto C43(DE3) competent cells and cultured at 37 °C in LB medium. Expression was induced with 0.5 mM IPTG at 23 °C for 3 h for ACOX1a and 30 °C overnight for ACOX1b. The cell pellets were lysed on ice by sonication in lysis buffer (20 mM NaPO4, 100 mM NaCl, 10% glycerol, 10 mM imidazole, 0.5 mM PMSF, 25 µM FAD, 1 mM benzamidine, 50 ug/mL lysozyme, 0.5% Tween-20, pH 8.0). Ultracentrifugation was employed to separate lysate supernatant and cell debris. The recombinant proteins were purified on HisTrapTM HP columns (Cytiva Sweden AB) by using an AKTA Pure system. The washing buffer is 20 mM NaPO4, 100 mM NaCl, 10% glycerol, 10 mM imidazole, 0.05% Tween 20, pH 8.0. The proteins were eluted with an imidazole gradient.

The human EHHADH coding sequence with a 6His tag coding sequence at 5′-end following a start codon was incorporated into the pET21a(+) plasmid. The EHHADH expression plasmid and a GroEL/ES (chloramphenicol resistant) plasmid were co-transformed onto C41(DE3) chemically competent cells. The cells were cultured at 37 °C in LB medium, and protein expression was induced by adding 0.5 mM IPTG when OD was 2. The expression was continued overnight with continuous shaking at 19 °C. The harvested cell pellet was lysed in 20 mM NaPO_4_, 100 mM NaCl, 10% Glycerol, pH 7.7 containing 0.5 mM PMSF, 1 mM benzamidine, 50 μg/mL lysozyme, 10 mM imidazole and 0.5% Tween 20. Ultracentrifugation cleared lysate supernatant was loaded on HisTrapTM HP columns (Cytiva Sweden AB) by using an AKTA Pure system. The unbound lysate components were washed away by washing buffer (20 mM NaPO_4_, 100 mM NaCl, 10% glycerol, 10 mM imidazole, 0.05% Tween 20, pH 7.7). The pure EHHADH protein was eluted with an imidazole gradient.

### 2.9. Enzyme Activity Assays

ACOX1a, ACOX1b or EHHADH were chemically succinylated by 0.1 mM succinyl-CoA in MSH buffer (210 mM mannitol, 70 mM sucrose, 5 mM Hepes, pH 8.0) with 5 mM DTT, 0.05% Tween-20 at 37 °C for 30 min. Desuccinylation was carried out in 50 mM Tris (pH 7.5) with 0.2 µg/ul SIRT5, 1 mM NAD, 2 mM MgCl2, 10% glycerol, 5 mM DTT, 0.05% TW-20. The buffers were changed to 50 mM NaPO4, pH 7.0 (ACOX1) or 50 mM Tris-HCl, pH 8.5 (EHHADH), with 10% glycerol and 0.05% Tween-20. Enzyme activity was then measured as follows. ACOX1 activity was assayed with 25 µM of acyl-CoA substrate following H_2_O_2_ production with Amplex Red as described [6,18]. In total, 1 µg of protein was added to 50 mM phosphate buffer pH 7.0 containing HRP (1 U/mL) and Amplex UltraRed (50 µM) at 30 °C. Reactions were started by the addition of acyl-CoA substrate, and fluorescence was monitored for 30 min. The EHHADH activity assay was conducted in 100 µL of 50 mM Tris, pH 8.5, with 0.05% Tween-20, 1 mM NAD, 5 µg EHHADH, and 10 µg ACOX1a or ACOX1b. Substrate concentration was 50 µM. The assay was carried out at 37 °C on a fluorescence plate reader (FluoStar Omega, BMG LabTech, Ortenberg, Germany), using an excitation wavelength of 340 nm and emission wavelength of 490 nm.

## 3. Results

### 3.1. Dicarboxylic Acid Feeding Recruits SIRT5 to Liver Peroxisomes

**Figure 1:**
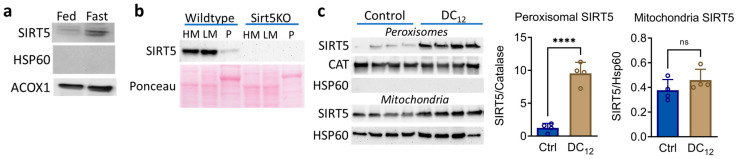
SIRT5 is recruited to liver peroxisomes by feeding mice DC12. (**a**) Liver peroxisomes were isolated from wildtype mice on standard diet under basal conditions (Fed) or after a 16 h fast (Fast). Immunoblotting shows the presence of SIRT5 in peroxisomes. Heat-shock protein-60 (HSP60) was used as a mitochondrial marker and acyl-CoA oxidase-1 (ACOX1) as a peroxisomal marker to demonstrate purity. (**b**) Liver heavy mitochondria (HM), light mitochondria (LM) and peroxisomes (P) were separated from wildtype and SIRT5KO mice on standard diet and blotted with anti-SIRT5. All lanes contain 16 µg of protein. Ponceau staining was used as loading control. (**c**) Wildtype mice were fed either standard laboratory diet (Control) or 10% *w*/*w* DC12 for five weeks. Peroxisomes and mitochondria were purified from *n* = 4 livers and blotted for SIRT5, catalase (CAT) as a peroxisomal marker, and HSP60. Densitometry was used to quantify the bands and generate bar graphs, which show means and standard deviations. ns, not significant; **** *p* < 0.0001 by Student’s *t*-test.

SIRT5 localizes in part to peroxisomes in cultured cell lines [5]; however, SIRT5 peroxisomal localization has not been directly demonstrated in vivo. Therefore, we used a density gradient centrifugation protocol to isolate pure mouse liver peroxisomes. Wildtype mouse liver peroxisomes, positive for the peroxisomal marker ACOX1 but negative for the mitochondrial marker HSP60, were immunoblotted for SIRT5. Indeed, trace amounts of SIRT5 were present in wildtype liver peroxisomes under basal conditions (Figure 1a). We estimated the basal amount of peroxisomal SIRT5 to be ~10% of the amount found in either the heavy mitochondrial fraction (HM) or light mitochondria fraction (LM) separated during the gradient centrifugation (Figure 1b). Fasting overnight increased the amount of peroxisomal SIRT5 (Figure 1a), suggesting that localization of SIRT5 to the peroxisomal compartment may be inducible under conditions that drive peroxisomal fatty acid oxidation (FAO). This led us to ask whether feeding mice dodecanedioic acid (DC_12_), a dicarboxylic peroxisomal FAO substrate, would recruit SIRT5 to the peroxisome. We previously showed that DC_12_ is chain-shortened in peroxisomes to succinyl-CoA, which then reacts with peroxisomal proteins to produce protein hypersuccinylation [6]. Indeed, mice consuming a diet containing 10% *w*/*w* DC_12_ (21% of calories) had an 8-fold increase in the abundance of peroxisomal SIRT5, whereas mitochondrial SIRT5 remained unchanged (Figure 1c). While we did not immunoblot unfractionated liver homogenates for SIRT5, our proteomics analysis (described below) indicated that DC_12_ feeding induced a ~25% increase in total hepatic SIRT5 protein. This increase was not statistically significant.

### 3.2. SIRT5 Knockout Mice Have Impaired DC_12_ Catabolism

**Figure 2:**
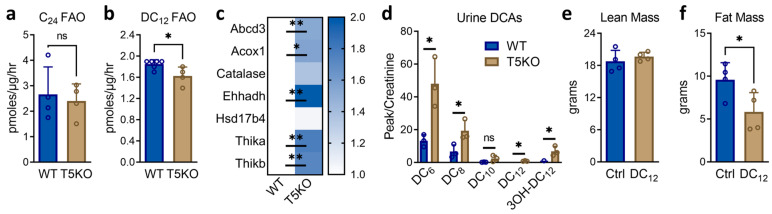
SIRT5 knockout mice have impaired DC12 catabolism. (**a**,**b**) Liver homogenates from wildtype (WT) and SIRT5KO mice (T5KO) fed DC12 at 10% *w*/*w* for five weeks were used to measure peroxisomal fatty acid oxidation (FAO) using 14C-C24 and 14C-DC12 as substrates. Results are normalized to total protein. (**c**) Mass spectrometry proteomics was used to quantify the expression of peroxisomal FAO proteins in liver after five weeks of DC12 feeding (N = 5). Heatmap represents fold-changes. Abcd3, ATP-binding cassette sub-family D member 3; Acox1, acyl-CoA oxidase-1; Ehhadh, enoyl-CoA hydratase/3-hydroxyacyl-CoA dehydrogenase; Hsd17b4, peroxisomal multifunctional enzyme type 2; Thika, 3-ketoacyl-CoA thiolase A, peroxisomal; Thikb, 3-ketoacyl-CoA thiolase B, peroxisomal. (**d**) Overnight urine samples (n = 3) in mice maintained on DC12 diet were used for mass spectrometry to quantify DC12 and partially shortened products of DC12 FAO. Data were normalized to urine creatinine. (**e**,**f**) SIRT5KO mice were fed standard diet (Ctrl) or DC12 for five weeks. EchoMRI was used to quantify total fat mass and lean mass (n = 4). All bar graphs represent means and standard deviations. ns, not significant; * *p* < 0.05; ** *p* < 0.01 by Student’s *t*-test.

Chen et al. [5] showed that succinylation increases the activity of ACOX1, the rate-limiting enzyme for peroxisomal straight-chain FAO, and conversely, desuccinylation by SIRT5 decreases ACOX1 activity. Therefore, we hypothesized that knocking out SIRT5 would enhance peroxisomal FAO via increased ACOX1 function. To test this, wildtype and SIRT5KO male mice were fed DC_12_ at 10% *w*/*w* for five weeks. Liver homogenates were used to assess the rate of peroxisomal FAO using ^14^C-DC_12_ and the very long-chain substrate ^14^C-C24. Opposite to our hypothesis, the rate of DC_12_ FAO was significantly reduced, not increased, by ablation of SIRT5, while C_24_ FAO was not changed (Figure 2a,b). These FAO assay rates were normalized to total liver protein. However, liver proteomics showed that the peroxisomal FAO machinery was upregulated by ~1.5 to 2-fold in DC_12_-fed SIRT5KO liver compared to DC_12_-fed wildtype liver (Figure 2c). If the FAO data were normalized to the abundance of the FAO machinery, then C_24_ FAO would be ~50% lower in SIRT5KO liver and DC_12_ FAO ~75% lower. In short, these data suggest that peroxisomal FAO is impaired in SIRT5KO liver and that peroxisomal enzymes are upregulated to compensate. In keeping with impaired D_12_ catabolism, DC_12_-fed SIRT5KO mice had elevated D_12_ intermediates in urine, including DC_12_, 3-hydroxy-DC_12_, DC_8_, and DC_6_ (Figure 2d). Despite this impairment, SIRT5 was not required for the reduced adiposity that we previously showed to be a hallmark of DC_12_ feeding [6]. SIRT5KO mice had ~40% less total body fat than SIRT5KO control-fed mice after five weeks on the DC_12_ diet (Figure 2e,f), although total body weight was not significantly different.

### 3.3. Knocking out SIRT5 Has Minimal Effects on the Peroxisomal Succinylome

**Figure 3:**
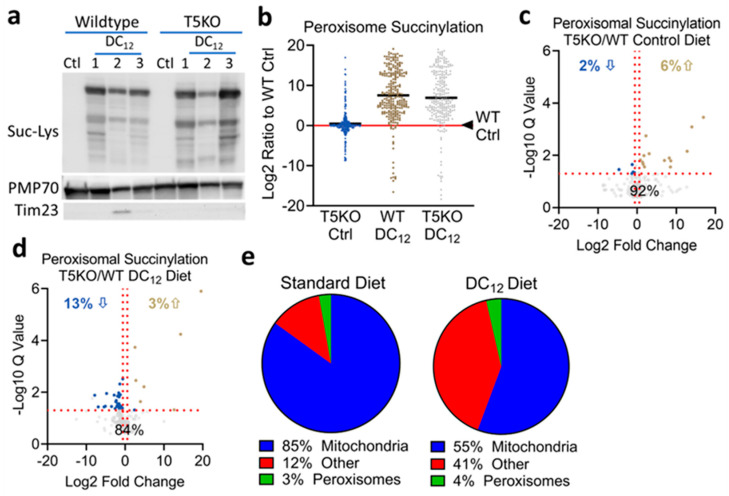
Knocking out SIRT5 has minimal effects on the peroxisomal succinylome. (**a**) Liver peroxisomes from wildtype and SIRT5 knockout mice (T5KO) on either standard diet (Ctl) or after feeding DC12 for 7 days were used for anti-succinyllysine immunoblotting. PMP70 was used as a peroxisomal marker (and loading control), and Tim23 was used as a mitochondrial marker to demonstrate purity. (**b**–**e**) *n* = 5 wildtype (WT) and SIRT5KO (T5KO) mice were maintained on standard diet or DC12 diet for five weeks. Mass spectrometry was used to quantify site-level changes in lysine succinylation in the peroxisome. A total of 253 unique succinylated peptides representing 45 peroxisomal proteins were quantified. Succinylated peptides were normalized to protein abundance. Each dot in panels (**b**–**d**) represents a succinylated peptide. In panel (**b**), the abundance of each peptide in WT mice on standard diet (Ctrl) was set = 1 (Log_2_ fold change = 0, red line). The other three groups are shown as log_2_ ratios to WT control. Panels c and d are volcano plots showing statistical significance for T5KO/WT succinylated peroxisomal peptide ratios on standard diet and DC12 diet, respectively. Gold dots represent putative SIRT5 target peptides that increased significantly upon SIRT5 ablation, blue dots are peptides for which succinylation significantly decreased upon SIRT5 ablation, gray dots had no significant change. (**e**) All putative SIRT5 target peptides, defined as peptides with significantly increased succinylation in the absence of SIRT5, sorted by subcellular localization.

Data presented in Figure 2 suggested that rather than enhancing peroxisomal function, knocking out SIRT5 may impair it. We next sought to probe the mechanisms behind the impairment by profiling SIRT5 actions in the peroxisome. To screen for SIRT5 interactions with peroxisomal proteins, we prepared liver homogenate from a SIRT5KO mouse, incubated the homogenate with His-tagged recombinant SIRT5, and used anti-His antibody to precipitate proteins complexed with SIRT5. Mass spectrometry identified 257 putative SIRT5 interacting proteins with > 10% peptide coverage (Supplemental Table S1). Of the 10 proteins with highest peptide coverage, three were peroxisomal. In total, 15 peroxisomal proteins were identified as SIRT5 interacting partners. Nearly all of these are involved in FAO (Table 1). Of particular interest were ACOX1 and enoyl-CoA hydratase/3-hydroxyacyl-CoA dehydrogenase (EHHADH), which are required for dicarboxylic acid chain-shortening [7]. Next, we examined the effect of SIRT5 ablation on the in vivo peroxisomal succinylome. First, we fed wildtype and SIRT5KO mice 10% *w*/*w* DC_12_ acutely for 7 days and purified hepatic peroxisomes. Anti-succinyllysine immunoblotting confirmed that lysine succinylation is very low in peroxisomes in mice fed a standard laboratory control diet (Figure 3a). DC_12_ dramatically increased succinylation in both wildtype and SIRT5KO peroxisomes. Densitometry analysis of the total succinylation signal normalized to peroxisomal PMP70 as a loading control showed a non-significant trend toward higher succinyl- ation in SIR5KO peroxisomes. To interrogate this further, we fed another cohort of mice the DC_12_ diet (or standard diet as control) for five weeks and used mass spectrometry to quantify changes in lysine succinylation at the residue level. A total of 253 succinylated peptides representing 45 peroxisomal proteins were quantified across both genotypes and both diets. To control for potential differences in gene expression, the abundance of the succinylated peptides was normalized to protein abundance. Using wildtype mice on standard chow as the benchmark, SIRT5KO mice had slightly increased peroxisomal lysine succinylation, with an average fold-increase of 1.3-fold (log_2_ of 0.43 in Figure 3b) across all 253 peptides. Wildtype mice on the DC_12_ diet had an average increase of 185-fold (log_2_ of 7.5 in Figure 3b), while SIRT5KO mice had an average increase of 121-fold (log_2_ of 6.9 in Figure 3b). This suppression of global peroxisomal lysine succinylation in DC_12_-fed SIRT5KO was unexpected. We further interrogated the mass spectrometry data set by separating the data by diet and statistically analyzing for SIRT5-dependent site-level changes in succinylation. Specifically, we sought to identify lysine residues demonstrating an increase in succinylation upon ablation of SIRT5, which would represent putative SIRT5 target sites in the peroxisome. On the standard control diet, 19 out of the total 253 succinylated peroxisomal peptides showed statistically significant differences, defined as q < 0.05 and fold-change > 1.5, between genotypes (Figure 3c). Of the 19 peptides, 14 were increased and 5 were decreased in SIRT5KO livers (Table 2). In the DC_12_-fed groups, there were a total of 41 out of 253 succinylated peptides that significantly differed between wildtype and SIRT5KO peroxisomes (Figure 3d). The majority of these (34 peptides) were significantly decreased, not increased, in SIRT5KO compared to wildtype (Table 3). Only three lysine residues were hypersuccinylated in peroxisomes across both diets in SIRT5KO mice—HSDL2 K295, NUDT19 K351, and EHHADH K355. Of these three proteins, only EHHADH plays a role in peroxisomal FAO. Of note, ACOX1, which was previously shown to be regulated by SIRT5 [5], had 17 quantified succinylated peptides. None were significantly different between wildtype and SIRT5KO under either diet. Most trended downward in SIRT5KO. Similarly, while EHHADH was hypersuccinylated on one lysine residue (K355) in the absence of SIRT5, 28 other lysine residues on EHHADH were statistically unchanged, with most trending downward in SIRT5KO. Together, these findings indicate that SIRT5 has minimal impact on the peroxisomal succinylome. To further illustrate this, we sorted all the significantly increased succinylated peptides in SIRT5KO liver by subcellular compartment. On standard diet, the putative SIRT5-targeted peptides were overwhelmingly mitochondrial (Figure 3e). On DC_12_ diet, there was a clear shift toward hypersuccinylation in “other” compartments upon SIRT5 ablation. The “other” category represents the cytoplasm, endoplasmic reticulum, nucleus, golgi, and plasma membrane combined.

### 3.4. SIRT5 Desuccinylates Recombinant ACOX1 and EHHADH but the Impact on Enzyme Activity Is Minimal

**Figure 4:**
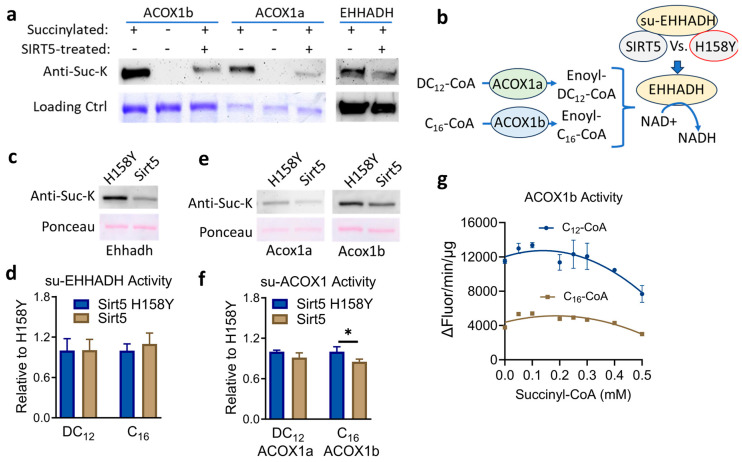
SIRT5 desuccinylates recombinant ACOX1 and EHHADH. (**a**) Recombinant human ACOX1a, ACOX1b, and EHHADH proteins were succinylated in vitro with 0.1 mM succinyl-CoA, and either mock-treated or treated with recombinant human SIRT5 and NAD+. The succinylation signal was detected by anti-succinylysine immunoblotting. The experiment was repeated with similar results. (**b**) Cartoon illustrating the EHHADH activity assay. Recombinant ACOX1 proteins were used to generate enoy-CoA substrates for EHHADH in a coupled assay. EHHADH activity was detected as the reduction of NAD+ to NADH. (**c**,**d**) Succinylated EHHADH protein was incubated with either inactive mutant SIRT5 (H158Y) or wildtype SIRT5. The resulting proteins were (**c**) immunoblotted for succinylation levels and (**d**) used for the activity assay illustrated in panel B. (**e**,**f**) Recombinant human ACOX1a and ACOX1b were succinylated and then incubated with either inactive mutant SIRT5 (H158Y) or wildtype SIRT5. The resulting proteins were (**e**) immunoblotted for succinylation levels and (**f**) used for a fluorometric ACOX1 activity assay that detects the formation of H_2_O_2_. (**g**) ACOX1b, which showed reduced activity after treatment with SIRT5 (panel **f**), was succinylated with increasing concentrations of succinyl-CoA and tested for activity with two monocarboxylic substrates, C12-CoA and C16-CoA, in triplicate. Data are plotted as means and standard deviations. * *p* < 0.05 by Student’s *t*-test.

In peroxisomes from DC_12_-fed mice, EHHADH and ACOX1, which are involved in DC_12_ chain-shortening [7], were the two most succinylated proteins with 29 and 17 quantified succinylated peptides, respectively. We, therefore, chose to study these two enzymes in more detail using in vitro methods pioneered by us and others in which recombinant proteins are succinylated chemically via exposure to succinyl-CoA [1,19,20]. Regarding ACOX1, there are two major splice variants, dubbed ACOX1a and ACOX1b, which are conserved across species [21,22,23]. We recently showed that only ACOX1a has appreciable activity with dicarboxylic substrates, even though the two isoforms differ by only 27 amino acids [6]. ACOX1b demonstrates a preference for longer monocarboxylic substrates like C_16_-CoA and C_24_-CoA [6]. Chen et al. studied the interaction between SIRT5 and ACOX1 but did not distinguish between the two splice variants. We, therefore, expressed and purified both recombinant human ACOX1a and ACOX1b, as well as human EHHADH. The proteins were incubated with 0.1 mM succinyl-CoA for 30 min to induce high-level lysine succinylation and then used as substrates for recombinant human SIRT5. All three proteins showed evidence of desuccinylation by recombinant SIRT5 in the presence of NAD^+^ (Figure 4a). Next, we asked whether this desuccinylation by SIRT5 impacts enzymatic activity. The physiological substrates for EHHADH are enoyl-CoAs, the products of the ACOX1 reaction. EHHADH is a bifunctional enzyme that first hydrates and then dehydrogenates enoyl-CoAs, reducing NAD+ to NADH in the process. Enoyl-CoAs are not commercially available. To test the effect of SIRT5 on the activity of EHHADH, we developed an approach in which chemically-succinylated EHHADH was incubated with NAD^+^ and either inactive mutant SIRT5 (H158Y) or active wildtype SIRT5 for 30 min. Then the succinylated and desuccinylated EHHADH proteins were buffer exchanged and mixed with either 1) recombinant ACOX1a and DC_12_-CoA or 2) recombinant ACOX1b and C_16_-CoA, in the presence of NAD+. In this coupled assay, the ACOX1 proteins converted their acyl-CoA substrates to enoyl-CoAs, which were then hydrated and dehydrogenated by EHHADH, with concomitant reduction of NAD^+^ to NADH, which was followed fluorometrically (Figure 4b). Desuccinylation of EHHADH by SIRT5 had no effect on EHHADH enzyme activity in this coupled assay, despite substantial desuccinylation by SIRT5 (Figure 4c). We similarly assessed the effect of SIRT5 on ACOX1a and ACOX1b activity, starting with chemically succinylated recombinant proteins and incubating with either SIRT5 or SIRT5 H158Y. SIRT5 had no effect on ACOX1a activity with DC_12_-CoA (Figure 4d). However, incubation of ACOX1b with SIRT5 did modestly reduce ACOX1b activity with C_16_-CoA, which agrees with the work of Chen et al. [5], who studied ACOX1 in cultured cells. In a final experiment, we incubated recombinant ACOX1b with increasing amounts of succinyl-CoA for 30 min prior to measuring enzymatic activity. At 0.1 mM succinyl-CoA, which is the concentration we used to succinylate ACOX1 proteins for the experiments in Figure 4d, there was a clear increase in ACOX1b activity with both C_12_-CoA, which is the optimum substrate for ACOX1b, and C_16_-CoA (Figure 4e). With exposure to increasing amounts of succinyl-CoA, the activity of ACOX1b declined, particularly with the optimum substrate C_12_-CoA.

## 4. Conclusions

This study demonstrated that SIRT5 can be recruited to the peroxisome under conditions that induce peroxisomal protein hypersuccinylation. SIRT5 was found to interact with and desuccinylate numerous peroxisomal proteins in vitro. However, deletion of SIRT5 had little effect on the peroxisomal succinylome in vivo. Despite the lack of change in the succinylome, SIRT5 knockout mice had signs of mild functional impairment of peroxisomal fatty acid oxidation. Further work is needed to understand the mechanisms by which SIRT5 is recruited to the peroxisome and the role it plays in tuning peroxisomal metabolism.

## 5. Discussion

Our findings confirm and extend upon the previous studies of Chen et al. [5], who showed that ACOX1 localizes partly to the peroxisome in multiple cell lines. Under normal, fed-state conditions, we saw a modest amount of SIRT5 protein within hepatic peroxisomes. Fasting, which is known to stimulate fatty acid ω-oxidation, dicarboxylic acid formation, and peroxisomal FAO, increased SIRT5 representation in peroxisomes. The fact the peroxisomal localization of SIRT5 was enhanced several-fold by feeding mice an exogenous dicarboxylic acid (DC_12_) suggests that the mechanism of recruitment is responding to the level of succinyl PTMs in the peroxisome. Future studies are needed to determine the mechanisms of SIRT5 peroxisomal recruitment. There are two known types of peroxisomal targeting signal (PTS) dubbed PTS1 and PTS2 [24,25,26]. PTS1 signals are variants of the canonical “SKL” motif at the carboxy terminus. PTS2 signals are variable and occur at either a terminus or internally on proteins. For SIRT5, Chen et al. found a putative PTS2 signal at the amino terminus, embedded within the mitochondrial targeting signal [5]. Our immunoblotting studies on purified organelles indicated that DC_12_ feeding induces a small, nonsignificant increase in mitochondrial SIRT5 while peroxisomal SIRT5 increases ~8-fold. This suggests that DC_12_ is not directing SIRT5 away from mitochondria into peroxisomes, but rather, increasing the total pool of SIRT5 protein. We did not, however, quantify cytosolic SIRT5 in the context of DC_12_. Future work will interrogate the balance of SIRT5 protein across mitochondria, cytoplasm, and peroxisomes, and seek to understand how this balance might respond to changes in PTMs within each compartment.

One unexplained phenomenon is why DC_12_-induced peroxisomal recruitment of SIRT5 has such a minimal effect on the peroxisomal succinylome. In mitochondria, protein succinylation is well-known to increase with SIRT5 ablation [2,27,28,29]. Indeed, our mass spectrometry survey found significantly increased succinylation in SIRT5KO liver on ~25% of all detected mitochondrial peptides, confirming previous studies. However, in the peroxisome, succinylation tended to be modestly suppressed in SIRT5KO liver after DC_12_ feeding, not increased. This is in keeping with reduced flux through the DC_12_ catabolic pathway. Despite having a significantly higher expression of key enzymes required for DC_12_ catabolism, such as ACOX1 and EHHADH, SIRT5KO liver homogenates showed a slower rate of ^14^C-DC_12_ FAO. Further, DC_12_ catabolic intermediates were higher in urine. The increased DC_12_ catabolic intermediates in urine, particularly 3OH intermediates, are similar to that reported in EHHADH knockout mice, which have a clear block in dicarboxylic acid FAO [30,31]. A slower flux of DC_12_ through the pathway would slow the rate of succinyl-CoA production from DC_12_, thereby lowering the overall level of peroxisomal protein succinylation. The fact that the acylating agent, succinyl-CoA, may have decreased in concentration inside the peroxisome upon SIRT5 ablation may have masked our ability to identify putative SIRT5 target sites on peroxisomal proteins. In other words, we would have expected succinylation to increase on SIRT5-targeted lysine residues in the absence of SIRT5, but an impaired ability to form the succinyl-CoA that is necessary to produce the increased succinylation serves as a confounding variable.

Regarding the mechanism behind reduced DC_12_ flux through SIRT5KO peroxisomes, we may only speculate. Given that ~95% of putative SIRT5 target sites in DC_12_-fed mouse liver are in the mitochondria or in “other” compartments such as the cytoplasm, the likely explanation is that an accumulation of succinyl PTMs in one or more of those compartments is responsible. One possibility is that mitochondrial FAO partly contributes to DC_12_ oxidation and that the reduced flux and accumulation of urinary DC_12_ metabolites in SIRT5KO mice is attributed to changes in mitochondrial FAO enzymes, not peroxisomal. While mitochondria are capable of oxidizing DC_12_ in vitro [16], other studies have shown that DC_12_ is strictly catabolized by peroxisomes [8]. The mitochondrial contribution to DC_12_ FAO remains controversial. Another possible explanation is that a cytosolic factor is responsible for the reduced flux of DC_12_ in SIRT5KO liver. For example, increased succinylation of acyl-CoA synthetases, which would be required to activate DC_12_ to coenzyme A, may suppress the rate of FAO. Alternatively, succinylation of fatty acid binding proteins (FABPs), which convey fatty acids to membrane receptors for organelle import, may be affected in the absence of SIRT5. A final area of speculation relates to the possibility that in wildtype liver, even though DC_12_ feeding increases the abundance of peroxisomal SIRT5 by ~8-fold, this pool of SIRT5 may be enzymatically handicapped by a lack of available NAD+. All sirtuins are NAD^+^-dependent [17]. The *K_m_* of SIRT5 for NAD+ is around 200 µM in vitro [18]. One biological explanation for this would be to prevent sirtuins from outcompeting metabolic enzymes for the shared NAD^+^ cofactor. In the peroxisome, each round of FAO chain shortening requires the reduction of NAD^+^ to NADH by EHHADH and HSD17B4, the two peroxisomal bifunctional enzymes. The *K_m_* of EHHADH and HSD17B4 for NAD^+^ has been estimated at ~5 µM [19], which is 40-fold lower than the *K_m_* of SIRT5 for NAD^+^. It is possible that when we feed animals DC_12_, the peroxisomal FAO pathway is constitutively activated, and under this context, the NAD^+^ is preferentially channeled into the chain-shortening of DC_12_ and SIRT5 is left inactive. Such a mechanism could conceivably explain the apparent lack of difference in the succinylome between wildtype and SIRT5KO mice on a DC_12_ diet—in essence, even the wildtype mice may be deficient in peroxisomal SIRT5 activity due to competition for NAD^+^. Future work is needed to investigate these intriguing possibilities.

## Figures and Tables

**Table 1:** Peroxisomal proteins detected in Sirt5 Co-IP.

Protein	Function	% Coverage
Uricase (UOX)	Oxidizes uric acid to 5-hydroxyisourate	86
Peroxisomal bifunctional enzyme type 2 (HSD17B4)	Steps 2 and 3 of β-oxidation	74
Peroxisomal bifunctional enzyme type 1 (EHHADH)	Steps 2 and 3 of β-oxidation	65
Acyl-CoA dehydrogenase 11 (ACAD11)	Step 1 of very long-chain β-oxidation	50
Peroxisomal 2,4-dienoyl-CoA reductase (DECR2)	Reduces double bonds, β-oxidation	49
Peroxisomal acyl-coenzyme A oxidase 1 (ACOX1)	Step 1 of β-oxidation	40
Catalase (CAT)	Degrades H_2_O_2_ to H_2_O and O_2_	33
ATP-binding cassette sub-family D member 3 (ABCD3)	Fatty acid transporter	23
Acyl-coenzyme A thioesterase 4 (ACOT4)	Cleaves CoA from succinyl-CoA	21
Non-specific lipid-transfer protein (SCP2)	Step 4 of β-oxidation	21
Peroxisomal sarcosine oxidase (PIPOX)	Metabolizes sarcosine & pipecolic acid	16
Lon protease homolog 2, peroxisomal (LONP2)	Degrades misfolded proteins	14
3-ketoacyl-CoA thiolase B (ACAA1b)	Step 4 of β-oxidation	13
Very long-chain acyl-CoA synthetase (SLC27A2)	Activates fatty acids into acyl-CoAs	12
Peroxisomal coenzyme A diphosphatase (NUDT7)	Hydrolyzes CoA moiety of acyl-CoAs	10

**Table 2:** Significantly altered succinylated peptides in liver peroxisomes on standard diet.

Protein	Lysine Residue	Log_2_ FC KO/WT
Increased Succinylation (q < 0.05)		
Hydroxysteroid dehydrogenase like 2 (HSDL2)	K481	16.9
Enoyl-CoA isomerase (ECH1)	K320	14.0
Enoyl-CoA isomerase (ECH1)	K76	12.8
Acyl-coenzyme A diphosphatase (NUDT19)	K351	8.6
Hydroxysteroid dehydrogenase like 2 (HSDL2)	K390	8.5
Alpha-methylacyl-CoA racemase (AMACR)	K204	8.2
Hydroxysteroid dehydrogenase like 2 (HSDL2)	K295	7.0
Prostaglandin reductase-3 (ZADH2)	K214	3.1
Dehydrogenase/reductase SDR family member 4 (DHRS4)	K106	2.3
Catalase (CAT)	K522	1.9
Peroxisomal 2,4-dienoyl-CoA reductase (DECR2)	K230	1.7
ATP-binding cassette sub-family D member 3 (ABCD3)	K6	1.5
Alanine--glyoxylate aminotransferase (AGXT)	K322	1.4
Peroxisomal bifunctional enzyme type 1 (EHHADH)	K355	0.8
Decreased Succinylation (q < 0.05)		
Dehydrogenase/reductase SDR family member 4 (DHRS4)	K99	−0.7
Catalase (CAT)	K98	−1.0
Peroxisomal trans-2-enoyl-CoA reductase (PECR)	K83	−1.3
Enoyl-CoA isomerase (ECH1)	K230	−1.3
2-Hydroxyacid oxidase 1 (HAO1)	K249	−4.6

**Table 3:** Significantly altered succinylated peptides in liver peroxisomes on DC_12_ diet.

Protein	Lysine Residue	Log_2_ FC KO/WT
Increased Succinylation (q < 0.05)		
Peroxisomal ATPase (PEX1)	K1205	14.3
Peroxisomal bifunctional enzyme type 2 (HSD17B4)	K50/K57	12.6
Alanine--glyoxylate aminotransferase (AGXT)	K411/K314	4.9
Hydroxysteroid dehydrogenase like 2 (HSDL2)	K295	4.0
Catalase (CAT)	K477	2.8
Peroxisomal bifunctional enzyme type 1 (EHHADH)	K348/K355	2.6
Acyl-coenzyme A diphosphatase (NUDT19)	K351	2.4
Decreased Succinylation (q < 0.05)		
Uricase (UOX)	K27	−0.6
Peroxisomal bifunctional enzyme type 1 (EHHADH)	K579	−0.7
Acyl-CoA dehydrogenase-11 (ACAD11)	K422	−0.9
Uricase (UOX)	K118	−1.0
Uricase (UOX)	K80	−1.1
Bile acid-CoA: amino acid N-acyltransferase (BAAT)	K346	−1.2
Peroxisomal sarcosine oxidase (PIPOX)	K104	−1.3
ATP-binding cassette sub-family D member 3 (ABCD3)	K260	−1.4
Bile acid-CoA: amino acid N-acyltransferase (BAAT)	K406	−1.4
Non-specific lipid-transfer protein (SCP2)	K26	−1.5
Peroxisomal bifunctional enzyme type 1 (EHHADH)	K527	−1.6
2-Hydroxyacid oxidase 1 (HAO1)	K353	−1.6
Peroxisomal bifunctional enzyme type 2 (HSD17B4)	K81	−1.6
Peroxisomal bifunctional enzyme type 1 (EHHADH)	K459	−1.6
Peroxisomal bifunctional enzyme type 2 (HSD17B4)	K57	−1.8
Dehydrogenase/reductase SDR family member 4 (DHRS4)	K99	−1.8
Peroxisomal bifunctional enzyme type 2 (HSD17B4)	K65/K68	−1.8
Hydroxysteroid dehydrogenase like 2 (HSDL2)	K87	−1.8
Acyl-CoA dehydrogenase-11 (ACAD11)	K90	−1.9
Peroxisomal 2,4-dienoyl-CoA reductase (DECR2)	K70/K71	−1.9
Peroxisomal trans-2-enoyl-CoA reductase (PECR)	K32	−1.9
Peroxisomal sarcosine oxidase (PIPOX)	K153	−2.1
Peroxisomal sarcosine oxidase (PIPOX)	K98	−2.1
Alpha-methylacyl-CoA racemase (AMACR)	K57	−2.3
Bifunctional epoxide hydrolase 2 (EPHX2)	K482	−2.4
2-hydroxyacyl-CoA lyase 1 (HACL1)	K21	−2.5
Enoyl-CoA isomerase (ECH1)	K230	−2.8
Peroxisomal coenzyme A diphosphatase (NUDT7)	K20	−3.5
2-hydroxyacyl-CoA lyase 1 (HACL1)	K238	−4.8
2-Hydroxyacid oxidase 1 (HAO1)	K309	−5.1
Acyl-CoA oxidase-2 (ACOX2)	K678	−5.4
Peroxisomal trans-2-enoyl-CoA reductase (PECR)	K17	−6.9
Acyl-CoA oxidase-2 (ACOX2)	K303	−7.8
NAD-capped RNA hydrolase (NUDT12)	K282	−8.4

## Data Availability

Raw data and complete MS data sets have been uploaded to the Center for Computational Mass Spectrometry, to the MassIVE repository at UCSD, and can be downloaded using the following FTP link: ftp://massive.ucsd.edu/v08/MSV000096026/ or via the MassIVE website: https://massive.ucsd.edu/ProteoSAFe/dataset.jsp?task=21320bba927a4e409db5416af1651591 (MassIVE ID number: MSV000096026; ProteomeXchange ID: PXD056506) (accessed on 21 November 2024).

## Supplementary Materials

The following supporting information can be downloaded at: https://www.mdpi.com/article/10.3390/biom14121508/s1. Table S1: SIRT5 interacting proteins identified by Co-IP.
